# Postural control telerehabilitation with a low-cost virtual reality protocol for children with cerebral palsy: Protocol for a clinical trial

**DOI:** 10.1371/journal.pone.0268163

**Published:** 2023-08-17

**Authors:** Valeska Gatica-Rojas, Ricardo Cartes-Velásquez, Alex Soto-Poblete, Luis Eduardo Cofré Lizama

**Affiliations:** 1 Tele-rehabilitation Technology Center and Neurosciences in Human Movement, Faculty of Health Sciences, Universidad de Talca, Talca, Chile; 2 School of Medicine, Universidad de Concepción, Concepción, Chile; 3 Institute of Mathematics and Physics, Universidad de Talca, Talca, Chile; 4 School of Allied Health, Human Services and Sports, La Trobe University, Victoria, Australia; Universitat de Valencia, SPAIN

## Abstract

**Objective:**

To establish the feasibility and effectiveness of a rehabilitation programme using low-cost virtual reality aimed at improving postural control in children with cerebral palsy—spastic hemiplegia. It also aims to compare the effectiveness of this programme under two delivery modalities, telerehabilitation (TR) and face-to-face (FtF).

**Methods:**

This is a registered randomized controlled clinical trial protocol (ACTRN12621000117819). Eighteen sessions of low-cost virtual reality therapy will be provided through both, FtF and TR modalities using a Nintendo Wii balance board. Each programme will last for 6 weeks and will consist of 3 sessions per week of 25 minutes each. Twenty patients diagnosed with cerebral palsy—spastic hemiplegia will be recruited for each group: FtF or TR (n = 40). Participants will be assessed at baseline, by the end of weeks 2, 4, and 6, and at weeks 8 and 10 (post-intervention follow-ups). The primary outcome will be the Center of Pressure sway area (CoP_area_); secondary outcomes will be standard deviation and velocity of the CoP in the mediolateral and anterior-posterior directions; tertiary outcomes will include the Modified-Modified Ashworth Scale for lower limbs, Modified Ashworth Scale for upper limbs, timed up-and-go tests, the timed one-leg standing and 6-minute walk test.

**Results:**

This study provides an assessment of the feasibility and effectiveness of an affordable rehabilitation programme using low-cost virtual reality aimed at improving postural control in children with cerebral palsy.

**Conclusion:**

The designed rehabilitation programme using low-cost virtual reality may improve postural control in children with cerebral palsy—spastic hemiplegia. The TR modality is likely to be as effective as the FtF modality. The TR programme has been designed to overcome access barriers to physiotherapy services for children with cerebral palsy in low-resource settings, remote areas, and in restricted mobility contexts.

## Introduction

Cerebral palsy (CP) is the most common condition treated by therapists in infant neurological units, and it is the most common cause of motor disability in children and adolescents [1, 2], with an increasing prevalence in developing countries [3]. According to the topographical classification, spastic hemiplegia (SHE) is one of the most prevalent types of CP [1, 4]. In SHE, the mobility of one side of the body is more affected, generating poor postural control during standing, leading to impaired performance in daily functional activities [5, 6]. To date, most studies have focused in developing interventions to improve upper-limb function in SHE, for example, virtual reality interventions, which further allow undertaking therapeutic training at home [7–9]. The development of therapeutic interventions aimed at improving postural control, on the other hand, has received less attention [10–12].

Efficient, inexpensive, remotely accessible, and engaging are some of the highlighted characteristics sought after in the development of novel physiotherapy interventions, particularly in children living in low clinical resources/access and remote areas [13–16]. The development of such interventions has been further emphasised since the recent COVID-19 pandemic, which interrupted non-essential rehabilitation services including those focusing on patients with CP and imposed mobility restrictions (lockdowns) [17]. An alternative to meet the aforementioned demands is the use low-cost technological tools and exergames to deliver telerehabilitation (TR) interventions. Additionally, the use of exergames in TR can help improving the quality and quantity of neurorehabilitation in the often-underserved population of children with CP. It is noteworthy, however, that previous studies suggest that a flexible dual-care model that integrates hospital/clinic and home-based TR rehabilitation is more efficacious than TR alone [13]. To date, two studies using TR in children with CP have shown promising results in terms of feasibility and effectiveness compared with standard face-to-face (FtF) interventions, however, these studies have not reported objective outcomes [14, 15].

Low-cost virtual reality exergames are well-accepted by children and adolescents due to their ludic, motivating and engaging features, which facilitate a nearly seamless incorporation into TR [16]. However, high-level evidence of the superiority of TR using low-cost virtual reality in comparison to FtF at clinical facilities interventions is still lacking. It is noteworthy that low-cost virtual reality TR also brings the possibility to standardize rehabilitation interventions, which is a pending task in neurorehabilitation [18]. By 2022, commercially available virtual reality systems cost around $500–1000 USD. Although cheaper options exist, they require to use smartphones, which increases the final cost. For this project, low-cost virtual reality refers to a system valued $100–200 USD, which in middle to high income countries is low [19, 20] in comparison to the out-of-pocket cost for transport to physical rehabilitation facilities. There are no brand-new systems for exergaming within the referred price range, nonetheless, refurbished Wii consoles, for example, can be found for <$200 USD.

This clinical trial protocol aims to establish the feasibility and effectiveness of a rehabilitation programme using low-cost virtual reality to improve postural control in children with CP. It also aims to compare low-cost virtual reality under two delivery modalities, telerehabilitation (TR) and face-to-face (FtF, control group). We hypothesize that a rehabilitation programme using low-cost virtual reality will improve postural control in children with CP and that this programme delivered using TR will be as effective as a FtF modality. The TR programme is designed to expand the coverage of physiotherapy services for children with CP in low-resource settings and in remote areas, but also to provide access to physiotherapy during restricted mobility as in the case of recent COVID-19-related lockdowns.

## Materials and methods

### Design

**Fig 1:**
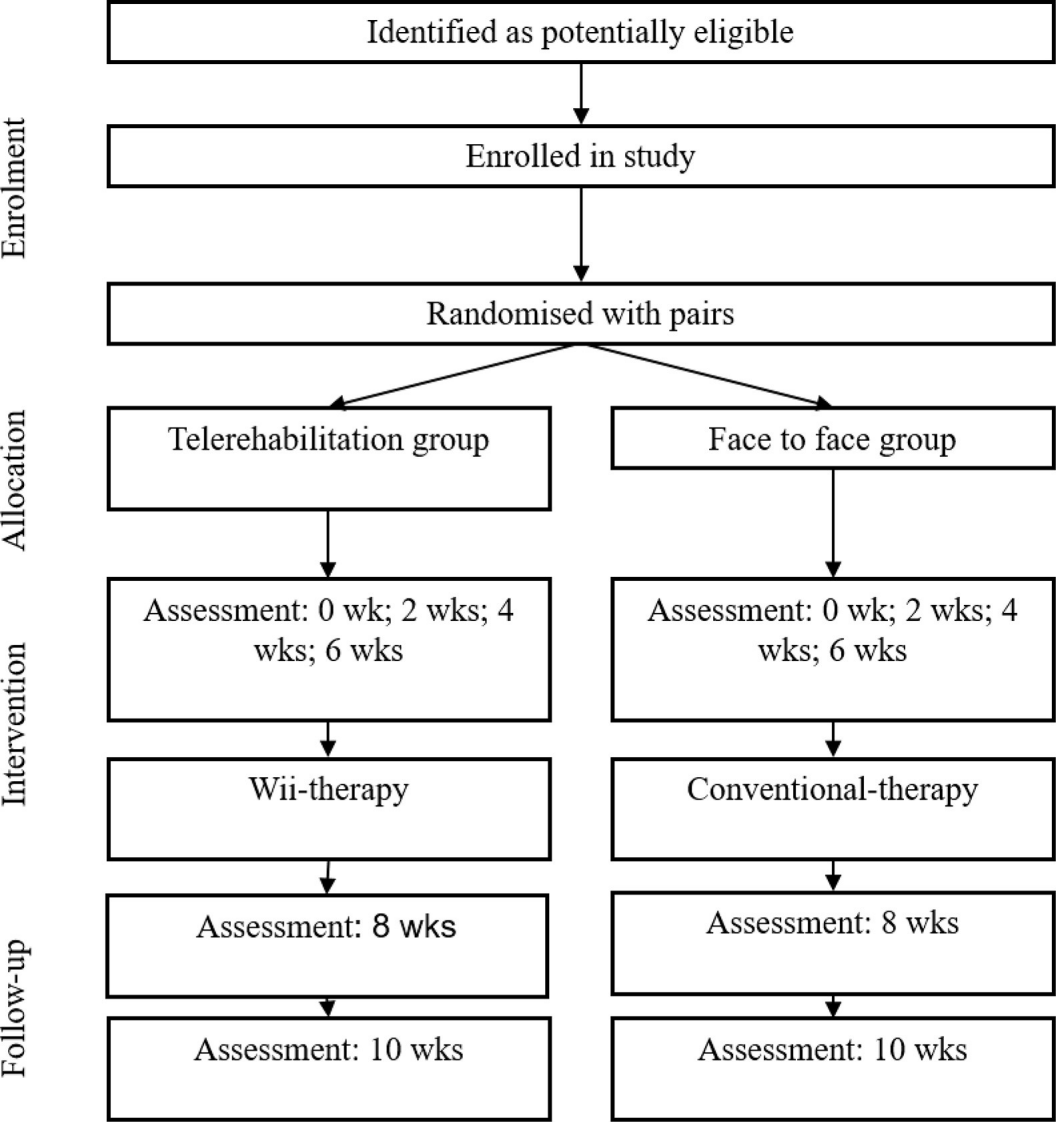
Flow chart according to the standard protocol items: Recommendations for interventional trials (SPIRIT). Participants, assessments and interventions. Through the two groups of the trial.

This is a double blinded (therapist and assessors), randomized, controlled trial designed following the Standard Protocol Items: Recommendations for Interventional Trials (SPIRIT), (Fig 1) [21].

The TR interventions does not require a therapist as in the FtF group, where therapist did not know about the participant’s involvement in the trial (therapist blinding). Assessors will not be able to identify the intervention group either. The trial adheres to the Declaration of Helsinki and the Chilean laws of rights and duties of the patient and research in humans. Ethical approval was obtained from the Ethics Committee of the University of Talca (Ref. No. 24–2018). This study was registered with the Australian and New Zealand Clinical Trials Registry (ACTRN12621000117819). Written informed consent will be obtained from parents and participants over 7 years old; verbal assent will be obtained from younger children.

### Study setting

This study will compare the effect of low-cost virtual reality in two distinct settings. The first setting will be clinical (Telerehabilitation Technology Centre and Neurosciences in Human Movement, Universidad de Talca, Chile) and in which low-cost virtual reality will be delivered in a FtF modality (control group) with therapist interacting directly with the patient. The second setting will be a remote facility with no therapist on-site for the delivery of low-cost virtual reality through TR. The default TR setting will be at-home, however, if a participant does not have adequate access to space and/or equipment, TR will be delivered at school, or rehabilitation centre. To note, in the latter cases (school and rehabilitation centre) no therapist will be involved in the TR delivery. All assessments will be conducted at the same facility in which the therapy will be delivered.

### Sample size

Using centre-of-pressure (CoP) sway area (CoP_area_) results from earlier studies [11, 22], to achieve an 80% statistical power with an alpha level of 0.05 and considering a standard deviation of 4.974 cm^2^ (after a 6-weeks intervention), we calculated a required minimum of 20 participants in each group (FtF and TR). This sample size includes a 5% attrition, which yields a total sample size of n = 40. Sample size calculation was performed using GRANMO (Institut Municipal d’investigació Médica, Barcelona, Spain). Once the investigative work has been carried out, the sample size will be verified again, and the estimation error will be adjusted. Feasibility of this study is ensured by our previous work with similar sample size, target population, outcomes, and funding [10].

### Eligibility criteria

The inclusion criteria will be as follows: (1) levels I or II on the Gross Motor Function Classification System (GMFCS) [23] and on the Expanded and Revised Gross Motor Function Classification System (GMFCS-ER) [24]; (2) aged 7 to 14 years (both sexes) with CP type spastic hemiplegia; and (3) having mild or no cognitive impairment. Exclusion criteria will include patients with other than CP neurological disorders, epilepsy, and uncorrected vision or hearing. Participants with access to a Nintendo Wii at home before the intervention commences will be also excluded.

### Interventions

This study will compare the effects of low-cost virtual reality delivered through FtF and TR modalities. The low-cost virtual reality will consist of using a Nintendo Wii balance board to play 4 exergames. In the FtF group, therapy will be provided by a physiotherapist with experience in the use of exergames for rehabilitation. In the TR modality, the intervention will be delivered by the patient’s parent or caregivers, who will be previously trained about how to deliver and assist exergames correctly and safely. In total, participants in both groups (FtF and TR) will receive 18 exergaming sessions divided in 3 sessions per week every other day (Monday, Wednesday, and Friday) over a period of 6 weeks. Each session will last for 25 minutes in which each participant will perform three series of exergames with 2-minutes rest in between games as we previously described [22]. The first two series will include Snowboard, Penguin Slide, and Super Hula Hoop. The first series will be played with arms on the side and the second one with hands on the waist to avoid the potential effect of training under one or the other strategy. The third series will be deep breathing in the Yoga game. During the first 2 weeks of training (first 6 sessions), patients will receive manual guidance and verbal instructions after which (7^th^ session onwards) only verbal instructions will be provided by the parents, caregiver, or physiotherapist.

In the Snowboard exergame patients will score by swaying (CoP displacement) in the anterior-posterior direction to swerve around flags whereas in the Penguin Slide patients will sway in the medial-lateral direction to score by catching fish while maintaining a pinguin within a continuously tilting platform. Finally, in the Hula Hoop game, patients will maintain hoops on the waist of an avatar by swaying in a (anti)clockwise circular pattern. The aim in the Yoga game will be to stand as quite as possible, first with eyes open and then with the eyes closed.

The designed low-cost virtual reality programme will be based on previous studies that have demonstrated similar interventions can effectively train postural control.^11^ For all participants in both groups (FtF and TR), adherence will be encouraged through telephone reminders. No modifications of the intervention protocols throughout the study are considered at this stage.

### Outcomes

Differences between the effects of FtF and TR using low-cost virtual reality on postural control will be assessed using posturographic measures. In brief, posturography assesses postural control through measures of the CoP sway, of which the most commonly used are sway area (CoP_area_), and trajectories and velocities in the medial-lateral (V_ML_) and anterior-posterior (V_AP_) directions [25]. These CoP variables have been shown to be sensitive in determining changes in postural control due to training in young adults [26], patients with post-stroke hemiparesis [27], patients with Parkinson’s disease [28],^,^ and children with CP [26–28].

Primary outcome in this study will be the CoP_area_, which has been shown to be a reliable and valid measure of postural control in different clinical and nonclinical populations [22–28]. The CoP_area_ is an overall measure of the ability of the balance control system to maintain a stable upright posture, to note, higher CoP_area_ values indicate poorer balance control. The CoP_area_ has been previously and extensively used as an outcome measure of interventions aimed at improving balance control in children with atypical motor control development [29–32].

The secondary outcome measures of this study will be the standard deviation (CoP_SD_), V_ML_ and V_AP_. The CoP_SD_ is a measure of the variability of CoP displacements whereas the CoP velocity is a measure of the ability to react to changes in posture with larger values indicating poorer balance control. The latter is due to late postural adjustments lead to large CoP excursion that demand rapid corrections to maintain stability [25]. If CoP_SD_ and/or V_ML_ and V_AP_ are reduced, this will be interpreted as a beneficial effect on the intervention.

**Fig 2:**
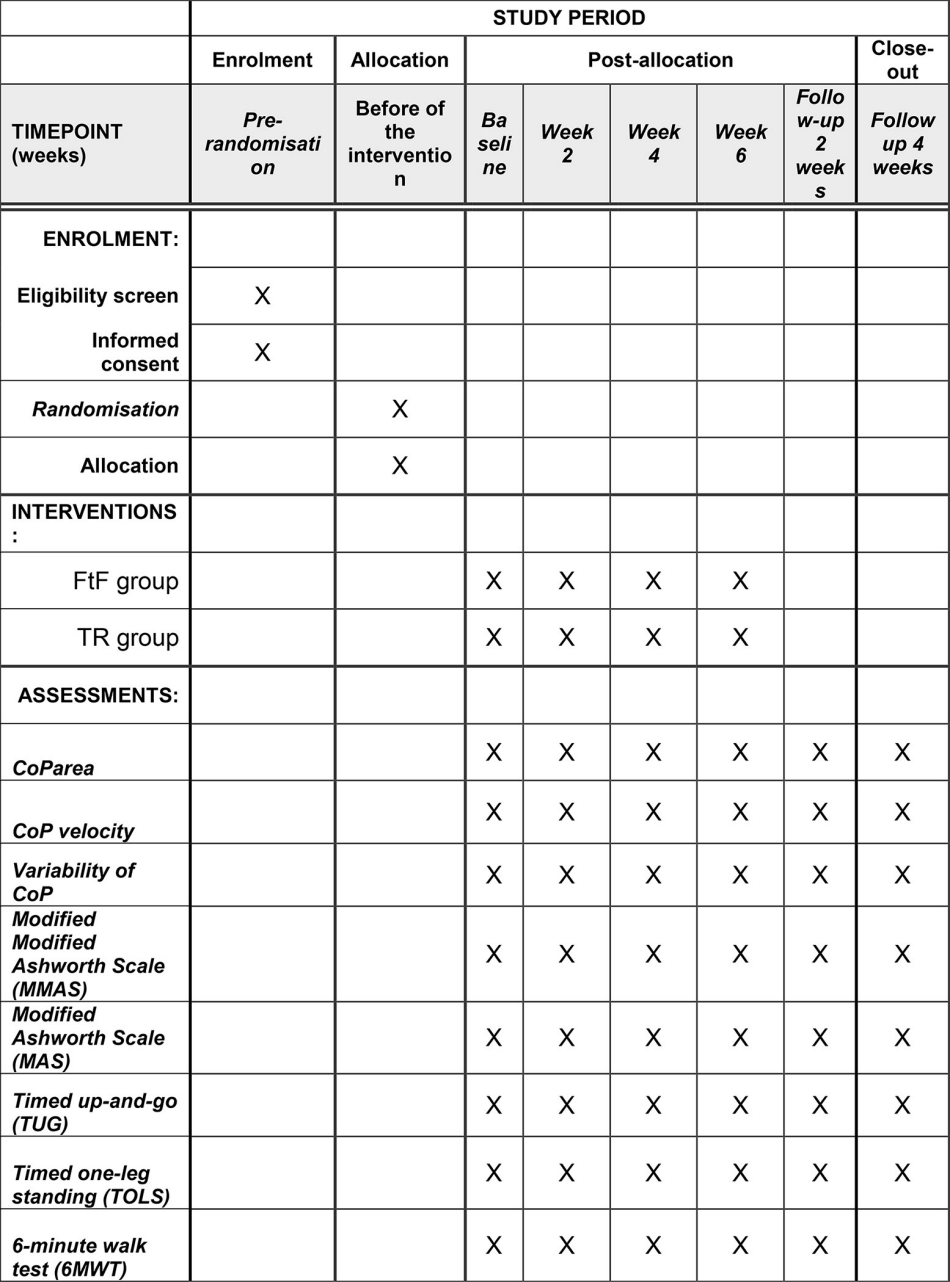
SPIRIT schedule of enrolment, interventions and assesments.

Tertiary outcome measures of this study will be the Modified Modified Ashworth Scale (MMAS) for lower limbs (ankle and knee flexor-extensor musculature), Modified Ashworth Scale (MAS) for upper limbs (elbow and wrist flexor-extensor musculature), timed up-and-go (TUG) tests, the timed one-leg standing (TOLS) and 6-minute walk test (6MWT). These are widely used clinical measures of spasticity (MMAS and MAS) and overall physical performance, which include some aspects of postural control (TOLS) and dynamic balance (TUG and 6MWT). Reductions in the MAS and MMAS spasticity scores, as well as TUG and 6MWT reduction and TOLS increase will be interpreted as beneficial effects of the interventions at a physiological and motor performance levels, respectively (Fig 2).

### Intervention timeline

Participants will be assessed at baseline, by the end of weeks 2, 4, and 6, and at weeks 8 and 10 (post-intervention follow-ups) using posturography and clinical measures.

### Recruitment

Children and adolescents with congenital SHE-CP will be recruited from the Rehabilitation Institute, Talca, Chile. This is an outpatient clinic that provides rehabilitation services to patients with neurological disorders including CP children. It is noteworthy, that patients from this centre will have little or no experience using a Nintendo Wii balance board. An experienced staff of health professionals, comprised of a physician and two physical therapists, will recruit participants according to the inclusion/exclusion criteria. Screening for potential participants will continue until the calculated sample size is achieved.

### Allocation

Participants will be randomly assigned to either control (FtF) or experimental group (TR) with a 1:1 allocation as per a computer-generated randomization schedule stratified by site and the baseline score of the Action Arm Research Test (ARAT; < = 21 versus >21) using permuted blocks of random sizes. The block sizes will not be disclosed, to ensure concealment. This statistical methodology is adjusted to the randomized and block design, allowing feasibility and affordability to carry out this clinical trial.

### Management

Participants will be informed of the group to which they will be randomly assigned at the beginning of the study. Participants in each group will be systematically asked about the content of the interventions received to maintain engagement and compliance. Due to the nature of the intervention participants cannot be blinded to allocation and since therapists are required only for the FtF group, no information sharing will occur between clinicians in both groups. Furthermore, since interventions and assessments will occur at very distant places in the FtF and TR, between groups contamination will be unlikely. There will be one independent assessor (clinician external to the research team) for each of the intervention groups. All data will be recorded in unidentifiable datasheets so researchers will be able to independently analyse data without having access to information about the allocation. To identify raw data well as processed posturographic metrics and clinical outcomes measures, all electronic files will be coded and backed up every week.

### Data collection

All measurements will be performed at the same time of the day, under the same conditions and by the same assessor at each time point. Posturographic (CoP) measures for both groups will be collected using an AMTI OR67 force platform (Watertown, MA, USA). The AMTI NetForce software (Watertown, MA, USA) will be used for the acquisition of moments and forces at 200Hz. Data will be recorded, coded, and stored on a personal computer. CoP variables will be calculated in Matlab R2012 (Mathworks Inc., Natick, MA, USA).

All primary and secondary posturographic measures will be assessed under 2 static and 4 dynamic conditions for 30 seconds each: (i) standing still with eyes open, (ii) standing still with eyes closed, voluntary sway in the mediolateral direction following a metronome set at (iii) 30Hz and (iv) 60Hz, and multidirectional sway while playing 2 different videogames that challenge (v) anterior-posterior postural control (snowboard) and (vi) medial-lateral postural control (pinguin). It is expected that all assessments, including clinical ones, will be completed within 30 minutes.

### Data analysis

Descriptive statistics will be used to summarize the demographic and clinical characteristics in both intervention groups, and unpaired t-tests and chi-square tests will be used for comparisons. For all outcome measures, the Shapiro–Wilk and Levene tests will be used to measure the normality and homogeneity of the variance, respectively. If the assumptions of normality are met, a t-test will be employed, otherwise we will use Mann–Whitney U test to determine differences between the FtF and TR groups. Also, if the assumptions of normality are fulfilled, we will employ a repeated measures analysis of variance (RM-ANOVA), otherwise will be use Friedman’s test. Post-hoc pairwise comparisons will be used to determine the interventions effect over time for each group (FtF and TR). Cohen´s *d* will be used to report effect sizes for the CoP variables. To allow repeated measures analysis, missing data will be adjusted by 1) replacing with the non-missing average values for each variable/week, and 2) performing multiple imputations. Both methods will be used to make sure that the results were concordant. For all analyses, a *p*-value ≤0.05 will be considered statistically significant. All statistical analyses will be performed using IBM-SPSS 20.0 (SPSS Inc., Armonk, NY, USA).

### Data monitoring

A permanent monitoring will be made by the clinical team, oversighted by the research team and the Ethics Committee of the University of Talca. All the possible adverse events will be informed immediately to the Ethics Committee of the University of Talca to determine trial’s modifications or cessation.

## Discussion

The aims of this clinical trial protocol are to establish the feasibility and effectiveness of a low-cost virtual reality to improve postural control in children with CP and to compare two low-cost virtual reality delivery modalities, telerehabilitation (TR) and face-to-face (FtF). We hypothesize that low-cost virtual reality will improve postural control and that both delivery methods will be equally effective. The results of this study will help to support remote access to rehabilitation services and deliver higher, compared to standard, intensity interventions. Further, this trial will support standardization and collaboration in the field of telerehabilitation by incorporating objective measures of postural control to already existing clinical measures.

### Benefits of remote access to rehabilitation services

We have previously shown that a FtF virtual reality intervention was effective at improving static and dynamic postural control and reducing spasticity in children and adolescents with spastic cerebral palsy [29–32]. Demonstrating that a TR is as efficient as a FtF is crucial to adopt TR as an alternative to expand the coverage and overcome access barriers to rehabilitation services for children with CP in low resources and remote areas. The TR intervention proposed in this protocol is less reliant on clinicians, which can help clinicians to prioritise appointments for those children with greater needs. Further, through direct involvement in the process, TR will empower patients and carers to (self-)guide rehabilitation and participate in selecting most engaging and motivating exercises (exergames) to maintain adherence to treatment. Patients and carers can also benefit from reducing the economic and time burden of travelling to/from clinical facilities to receive physiotherapy treatments [18, 19]. In the recent COVID-19 pandemic context, a TR modality can offer a greatly needed alternative to avoid interruption of the rehabilitation process when mobility restrictions are in place (i.e. lockdowns).

### Higher intensity rehabilitation: greater benefits?

High-intensity physiotherapy is necessary for optimizing motor learning and improve physical skills in patients with CP [33]. Regrettably, children and adolescents with CP typically receive limited physiotherapy sessions, which implies a lost opportunity to improve the functionality and quality of life of these patients during a critical period of their lives. It is important to note that the neural maturity of sensory systems responsible for the control of postural balance is sequential and dependent on age, reaching full maturity at 15–16 years [34, 35]. Therefore, providing as much rehabilitation time as possible during this age period is essential to generate motor function improvements that will profoundly impact their quality of life during adulthood. To our knowledge, the long-term benefits of high intensity physiotherapy delivered through telerehabilitation have not yet been stablished. The TR proposed in this protocol can offer a methodology to explore its potential benefits and determine the impact in patient’s daily life over the long-term.

### Exergames as a tool for standardization and collaboration

There are several other medical and environmental factors that may accompany the type of CP and affect postural control. Such factors may demand tailored interventions, which are less likely to be accessible in low-resource and/or rural settings. Often seen as a downside for comparisons across studies, the heterogeneity and plethora of interventions available for CP patients may help in determining the most suitable intervention for patients with more specific needs [36–39]. Low-cost virtual reality exergames offer the possibility of objectively standardizing rehabilitation interventions to be better replicated in different settings yet still allowing certain degree of flexibility (i.e. difficulty levels) to be tailored to each patients’ needs. This manipulation (setting adjustments) can help enhancing multi-sensory interactions to increase motivation and engagement [40]. Since most exergames re-purposed for rehabilitation are created for worldwide markets, this offers the possibility for multisite interventions and collaborations across the globe [41, 42]. However, the optimal number of sessions, duration, sequence, games, intervals, amongst others, are yet to be explored. In this line, a recent systematic review on the effectiveness of virtual reality in CP patients found limited confidence in effect estimation for utilising virtual reality in this population [18].

Home-based therapies for patients with CP have shown to be feasible; however, previous studies have used a variety of methods and strategies, making comparisons across studies and overall conclusions of effectiveness difficult to stablish [43]. Although, previous research has only provided with low-quality evidence [44], two studies have shown promising results [13, 14]. Thus, further high-quality trials, as the one proposed in this protocol, are necessary to elucidate the benefits of virtual reality interventions.

A low-cost virtual reality, such as the Nintendo Wii device allows greater accessibility and transferability to patients with CP at neurological care centres than, to date, rehabilitation customised systems [45, 46] The Nintendo Wii and its peripheral balance board have been extensively used and studied as a training tool to improve standing balance in the elderly, adults with total knee replacements, post-stroke patients, and patients with Parkinson’s disease [9, 47–50]. Despite being discontinued in 2014, the Nintendo Wii continues to be a relevant tool for neurorehabilitation research and has been successfully used to treat patients with CP [51–53]. The latter may be of interest for manufacturers seeking to develop similar systems for neurorehabilitation of CP or paediatric populations.

### Objective and clinical measures: A comprehensive picture

This clinical trial protocol includes a set of comprehensive posturographic and clinical assessments that will help determining the effects of the FtF and TR interventions beyond postural control (e.g. walking and spasticity). We decided to use CoP variables as primary and secondary outcome measures as they have shown to be sensitive to the effects of physiotherapy interventions [54, 55]. The CoP_area_, our primary outcome measure, quantifies the whole body oscillations when standing; greater values indicate poorer balance control as CoP gets closer to the limit of stability, which demands rapid stabilization responses expressed in greater CoP velocities, our secondary outcome measures (V_ML_ and V_AP_). CoP_area_ reductions, as an indication of improved balance, has been found to be sensitive to rehabilitation intervention [22], whereas V_ML_ and V_AP_ have shown to be reliable measures of postural control in ageing populations.[55]

Previous studies have found that posturographic (CoP) measures are poorly correlated with clinical measures[56, 57], however, this may indicate that both sets of outcome measures capture different dimensions or components of the mobility problems associated to motor performance in CP patients. By including clinical assessments as tertiary outcome measures, we will be able further explore these correlations and determine, for example, if spasticity measures mediate or moderate functional outcomes during postural control performance in standing trials. In this study we will utilise a laboratory-grade forceplate to record CoP displacements and obtain CoP measures using a customised software, however, less expensive options using the same Wii balance board also exist [51].

### Meeting patient’s demands in a changing environment

This will be the first study to determine the comparative effectiveness of two delivery modalities, telerehabilitation (TR) and face-to-face (FtF), of a six-week exercise programme using a Nintendo Wii balance board to improve standing balance in children and adolescents with CP—spastic hemiplegia. This low-cost virtual reality programme has been primarily designed to meet the much-needed demands for remote access to physiotherapy services for patients with CP in low-resource settings. The developed programme will also help clinicians and patients to adapt and continue with the rehabilitation process even when physical access to services is restricted. Further, demonstrating the effectiveness of the TR, will be the first step for further research using higher intensity TR interventions that may have an even greater effect in motor control capabilities of CP patients than conventional therapies and that may have a long-term effect on their quality of life, social engagement, and participation.

## Supporting information

S1 Checklist (DOC)

S1 File (PDF)
