# Supplementary material for: Postural control telerehabilitation with a low-cost virtual reality protocol for children with cerebral palsy: Protocol for a clinical trial
Source: PLoS One. 2023 Aug 17;18(8):e0268163. doi: 10.1371/journal.pone.0268163 (PMC10434878; doi:10.1371/journal.pone.0268163)
Supplement: S1 File — (PDF) [file pone.0268163.s002.pdf]

# FIC FUNDING
# Maule Regional Government

Title: Implementation of an exercise program with virtual reality via telerehabilitation for patients with cerebral palsy type spastic hemiplegia and for older adults.

INSTITUTION: Universidad de Talca, Talca, Chile

## I. Objetives[1]

**Implement an exercise program with Nintendo Wii Balance Board via telerehabilitation for patients with cerebral palsy (CP) type spastic hemiplegia and older adults.**

**Specific objectives:**
1.- Develop via telerehabilitation the exercise program with Nintendo Wii Balance Board in the homes of patients with CP type spastic hemiplegia in rural areas of the Maule region, in neurological centers in the Maule region, a school and an older adults community center in the city of Talca.

2.- To compare the effectiveness of the exercise program with the Nintendo Wii Balance Board in a face-to-face clinical context with the therapist and with respect to a distance format via telerehabilitation, both in children-adolescents with CP of the spastic hemiplegia type and in older adults.

3.- Publish the results to regional and national health entities in charge of rehabilitation and relevant scientific communities.

4.- Generate advanced human capital through the development of postgraduate theses.

5.- Train and train older adults monitors to contribute to the transfer of the virtual reality exercise program to other older adults community centers in the city of Talca and Maule region.

[1] Describa objetivo general y específico.

## II. Background

**Worldwide technology of exercise programs for cerebral palsy and for the elderly**

Despite all the efforts made so far, there is no remote application -via telerehabilitation- of a protocolized exercise program or low-cost therapy ($<180 thousand and with free software) that uses a virtual reality interface to improve motor disability in CP and functionality in older adults, in the world. The concept of remotely monitoring patients or users in developed countries such as Australia and the United States (Truter et al, 2014) has been through telemedicine, a concept that is similar to the previous one, but only seeks to monitor the health status of patients. by means of a videoconference investigating the state in general, making certain specific evaluations such as range of movement, joint position and posture in general (Mani et al., 20016; Richardson et al., 2016). A similar case is for the neurological area, specifically patients with sequelae of a cerebrovascular accident (CVA) (Veras et al., 2016).

On the other hand, a recent meta-analysis compared the effects of remote with face-to-face (therapist-patient) rehabilitation therapy in a variety of musculoskeletal conditions, demonstrating that the real-time version of telerehabilitation was equivalent to the face-to-face (SMD MD 0.14, 95% CI -0.10-0.37, I2 = 0%) seen in improvement in physical function and pain (Cottrell et al., 2016).

Regarding telerehabilitation, Move it to improve it (MitiiTM) is an online training concept that is currently marketable and used by developed countries (mainly Australia) (Boyd et al., 2015). The MitiiTM has been validated to improve functionality in the upper limb and certain daily living skills in children affected by acquired brain damage (Boyd et al., 2015), according to term and without cognitive impairment. Therefore, the MitiiTM is not transferable to children and adolescents with CP in the world, where one of the main causes is prematurity accompanied by low birth weight, in addition, most children have associated cognitive impairment ranging from mild, moderate or severe (Cans, 2000; Johnson, 2002). From a technological point of view, since the Mitti uses a Kinects camera, it therefore captures all the movements (ideal and compensations or non-ideal) of the patient, including those of the relative who is close to the camera's viewfinder. This nullifies the possibility that a family member can closely guide the session

with the patient, which in many cases is required for children and adolescents with CP.

We have demonstrated the beneficial effects of virtual reality on improving postural balance in children and adolescents with CP (Fonis Sa112018) (Gatica et al., 2013a; Gatica et al., 2014b; Gatica-Rojas et al., 2016c). , mainly in the spastic hemiplegia type (Gatica-Rojas et al., 2016d). The exercise protocol was tested, described under the CONSORT guideline, and internationally registered (RBR-3sc9zc) through a randomized clinical trial with a power of 80%, an alpha of 0.05, and a minimum expected difference of 21.5 cm2; the sample consisted of 36 participants (16 in each study branch) (Table 1, right column). One branch received standard therapy and the other virtual reality therapy (NWBB as interface) (Gatica-Rojas et al., 2016d-accepted). This investigation demonstrated that 6 weeks (25 minutes each session/3 times/week) of the NWBB exercise program is more effective than standard therapy, and spastic hemiplegia-type CP improved balance or postural equilibrium more than spastic diplegia-type CP.

In the case of older adults (AM), only distance rehabilitation via telerehabilitation in arthroplasty or total knee replacement has been tested (Tousignant et al., 2011), as well as to provide rehabilitation services to patients in their homes. when they are discharged from a hospital (Tousignant et al., 2006).
On the other hand, a very important aspect is the physical abilities that are compromised over the years. In this sense, one of the most compromised physical abilities in older adults and that directly affect their health: is balance or postural balance, a generic term that describes the dynamics of body posture to prevent falls and is related to the inertial forces that They act on the body and body segments. The maintenance of postural balance is considered an essential requirement for functional independence, health status and quality of life in older adults; and its increase or deterioration constitutes a primary risk factor for falls, leading to a hip fracture which has high morbidity and mortality in older adults in the world and in Chile (Shkuratova & Taylor, 2008; Casen 2013). On the other hand, the benefit in different motor skills, including balance, of virtual environments through the use of an interface such as the Nintendo Wii console has been demonstrated in conditions of cerebral palsy, Parkinson's disease, post-stroke and older adults. healthy (Gatica & Méndez, 2014e). In 2010, the group of Gatica et al. (through 2 internal projects of the Research Department of the University of

Talca) tested an exercise protocol with NWBB in a population of twenty healthy older adults with loss of balance or postural balance. with a duration of 25 minutes each session, 3 times a week, for 8 weeks. The results of this investigation indicated that postural balance was significantly improved at 4 weeks, reaching a maximum at the sixth week of training (Gatica et al., 2010f).

To our knowledge, there is no virtual reality exercise program via telerehabilitation and monitored in real time for children and adolescents with CP and older adults, as the main outcome is to improve balance and postural control. Therefore, implementing it will allow solving and caring for people with disabilities, mainly children and adolescents with CP of the spastic hemiplegia type and the elderly population. In both, balance and postural control will be improved, directly contributing to their activities of daily living, improving their quality of life and that of their families. In addition, the possibility of falls in older adults is reduced and therefore its concomitant complication, hip fracture, which has a high morbidity and mortality in Chile. By improving these factors, health care demands, economic costs associated with these demands to the different health services, and costs associated with patients in their respective transfers to care centers are reduced.

## Methodology[2]

**-Design:** Two parallel clinical trials, one for patients with CP spastic hemiplegia (first stage) and another for the elderly (second stage). Parallel, since each clinical trial will make a comparison between the face-to-face version with the therapist and the remote version in real time via telerehabilitation (Figure 2).

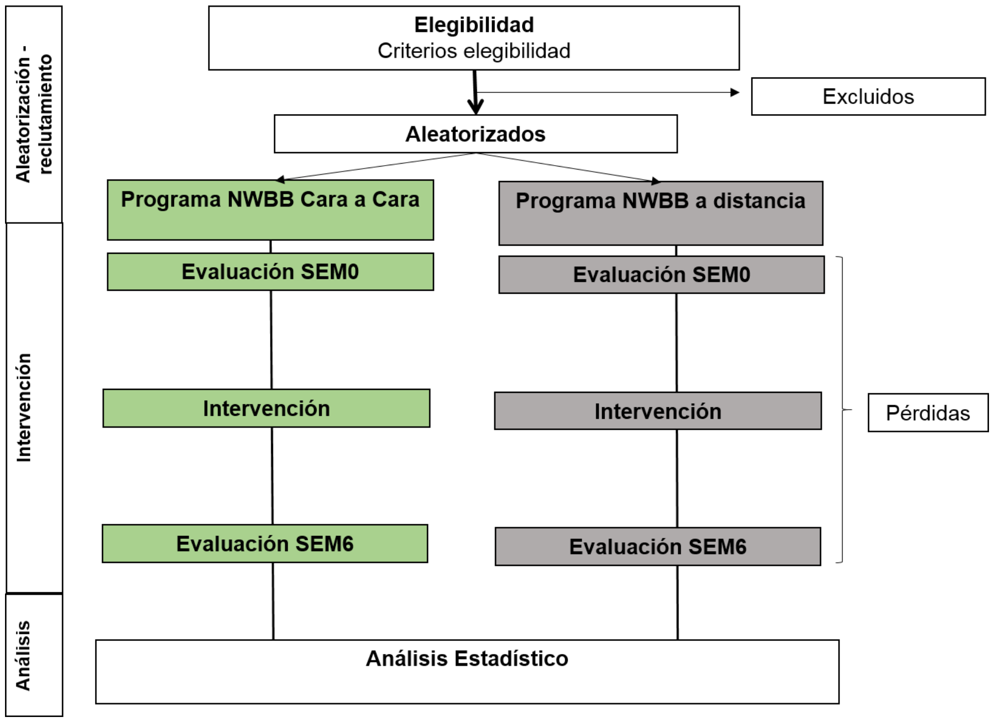


**Figure 2:** General model of randomized clinical trial according to the CONSORT guideline, for both study populations.

[2] Describir la metodología de trabajo para la obtención de los resultados esperados.

**- Population and participants:**
Clinical trial 1.- The participants will come from Teletón del Maule and the UP Neurorehabilitation Center (Curicó). Maule Telethon contains in its population the patients of the Enseña Special School. The population of Teletón Maule is close to 1700 patients aged 0 to 24 years. Based on the statistical records (admittances) of the Teletón Maule and Neurorehabilitation UP centers, the total population of children and adolescents with CP of the spastic hemiplegia type is 69 patients. Participants will be invited through informative talks that will take place in each of the establishments. Said talks will be delivered by the Director of this project addressed to the parents/legal guardians/guardians and the directors of the establishments.

**Selection of participants**

*Neurology:*

As indicated in the project, the patients of Teletón Maule (contains in its population the patients of the Enseña Special School) and the UP Neurorehabilitation Center have a database of patients updated every year (new patients, discharged, transferred and in the unfortunate case, the deceased are also recorded). Both centers have a CUBO OLAP system or similar, which corresponds to a database of statistical records. This system provides a global classification of the patients who attend these centers such as age, topographic diagnosis, corrective orthopedic surgeries performed, botox application, among others. These databases are used by rehabilitation centers to order forms of care, that is, by "areas" for example: cerebral palsy, types of cerebral palsy, neuromuscular diseases, types of neuromuscular diseases, ages of patients in each condition. / pathology, etc. However, in no case does this system correspond to a patient's clinical record; document that contains historical and specific background of the evolution of the patient in particular, in addition to socioeconomic, cultural and legal factors in some cases, as appropriate.

Clinical trial 2.- The Maule region has 170,248 AM, where the city of Talca has about 22,000 older adults (OA). AM participants will come from Club Los Álamos in the city of Talca, which has 48 healthy older adults (without pathological aging). Participants will be invited through informative talks that will take place in the OA Club. Said talk will be previously agreed with the Club's directive to define a date and time. The Director of this project will give

the talk in an expository, schematic way and will use a non-technical vocabulary.

*Older Adults:*

In the case of the Los Álamos older adults club, there is also a statistical record of the members (new, old, retired and older adults who have died), which is updated every year by the Club's board of directors or by this research team. after the terms of the community intervention project. In the event that it was not updated at the time the recruitment began, during the informative talk on the invitation to participate -indicated in the original project- older adults will be consulted individually for the data.

Then, in these databases (neurology and older adults), you can enter the list of eligibility criteria, and in this way filter the information that is in them, obtaining the potential participants who could -if so their consent or assent - participate. If in the supposed case any eligibility criteria were missing due to not being registered, this criterion must be consulted personally and privately by the participant (neurology and the elderly).

Research Briefing Note vs. Eligibility Criteria: It is likely that in the briefings (neurology and older adults, separately) attendees who do not meet the eligibility criteria are likely to be encouraged to participate. In this case they will be incorporated to prevent potential frustrations or other feelings related to it, however their measurements will not be considered for the analysis of the investigation.

**- Sample selection criteria:**

Clinical trial 1.-

Inclusion criteria:

1. Volunteer patients with CP type spastic hemiplegia (men and women)
2.- Age from 7 to 14 years
4. Mild or moderate motor functional impairment (GMFCS*).
5. Without mental retardation and/or mild mental retardation (visa based on medical evaluation).

*GMFCS: Gross Motor Function Classification System

Exclusion criteria:

1. Neurodegenerative pathologies
2. Vestibular alterations.
3. High dose of anticonvulsants.

4. Lower limb surgeries in the last 10 months.

***To distinguish the exclusion criterio***

-Neurodegenerative pathologies, refers specifically to neuromuscular diseases, which is a huge family, among the most common are Duchenne muscular dystrophy and Becker muscular dystrophy. This criterion will be detected by means of filters in the CUBO OLAP database or similar.

-Vestibular alterations, only those that affect the inner ear, specifically the labyrinth (utricle, saccule and semicircular canals). They directly affect people's balance and postural control. This criterion will be detected by means of filters in the CUBO OLAP database or similar.

-High doses of anticonvulsants, high doses directly affect the central nervous system. This criterion can be detected by means of filters in the CUBO OLAP database or similar or in the case that it is not updated in the database (data that varies more than once a year, depending on the evolution of the patient) should be consulted directly with the patient or legal guardian (father, mother or relative).

-Surgeries in the lower limbs in the last 10 months, refers to corrective orthopedic surgeries in the hip, knee or ankle joints. These surgeries are commonly applied to patients who during their development suffer bone or joint deformations as a result of the condition/pathology or its consequences, such as having high spasticity (increased skeletal muscle tone due to upper motor neuron damage). This criterion will be detected by means of filters in the CUBO OLAP database or similar.

Clinical trial 2.-

Inclusion criteria:

1. Men and women from 65 to 75 years of age
2. Use of corrected occasional or permanent lenses
3. Volunteers-informed consent process

Exclusion criteria:

1. Abbreviated Mini Mental State Exam <14 points
2. Neurological, vascular, vestibular, muscular disorders or any disorder that affects standing postural stability.
3. Three or more reports of falls in the last year.

4.- Execution of another modality of physical exercise during this investigation.

***To distinguish the exclusion criterio***

-Abbreviated or Mini-Mental State Mini-Examination of the Application Manual of the Preventive Medicine Exam for the Elderly, refers to the evaluation of the current cognitive state and the cognitive reserve acquired through formal education. It is an examination or evaluation that is usually carried out on the elderly population in our country in primary care health establishments. Examination carried out by any professional in the health area (nurses, kinesiologists, etc.). Your score is included in the card or notebook of the elderly, therefore from this document you can know its value. However, this evaluation will be carried out by the professional clinical team participating in the project (specifically a kinesiologist) prior to beginning the investigation. In the manual, a score ≤13 is referred to as altered, sufficient data (other symptoms exist or not) to be referred for clinical evaluation by a doctor. However, for this research team, given its experience in the subject, it has established a score of at least 14 to participate in the research described. If this exam yields a score ≤13 in any LO, immediate referral will be made, as follows: (i) request for medical control at the CESFAM that belongs to or regularly attends, or (ii) request for private medical evaluation . In any of these cases, a family member of the OA will be instructed to decide which option they prefer (i or ii) and to accompany the OA in the process.

-Neurological, vascular, vestibular, muscular disorders or any that affect postural stability while standing. Older adults have a high prevalence of these disorders, which are a broad family of conditions/pathologies, as I indicated on Wednesday, September 5, at the CEC meeting for the purpose of correctly addressing the post-review questions of this project. This project will directly consult the older adults for each of these disorders, if any, possible voluntary participants to consider in the data analysis with levels that do not affect standing stability – under the postural control construct declared in the original project ( item materials and method)- measured with a force platform for 60 seconds. and with a clinical test of standing on one foot for as long as it can be done. Note: keep in mind the note of the research informative talks versus eligibility criteria, stated in point 2.

-Three or more reports of falls in the last year: older adults will be consulted individually, live (session at the OA Club) and privately prior to starting the investigation.

-Execution of another modality of physical exercise during this investigation: the participating OAs are considered as healthy OAs (with controlled

pathologies) and active (non-institutionalized).

**-Sample size:**

The calculation of the sample size carried out was to estimate a population mean, this was an error of interpretation of the main objective by the statistician. However, the detection of this error is appreciated, which is rectified as follows: the calculation to compare population means of two independent trials, ensuring a maximum difference $\delta$.

The hypotheses to be tested are the following:

$H_0: \mu_A = \mu_B \qquad H_0: \mu_A - \mu_B = 0$

$H_0: \mu_A \neq \mu_B \qquad H_0: \mu_A - \mu_B \neq 0$

Where:

$\mu_A$: is the population mean of the first trial

$\mu_B$: is the population mean of the second trial

Sample size for difference of two independent means:

$$n_0 = 2\left[\frac{\left(Z_{\alpha/2} + Z_\beta\right)\sigma}{\delta}\right]^2$$

Where,

$\alpha: error\ tipo\ I$

$\beta: error\ tipo\ II$

$\sigma: Desviación\ estándar\ poblacional$

$\delta: diferencia\ mínima\ a\ detectar$

References: Jay L. Devore (2008), *Probabilidad y Estadística para Ingeniería y Ciencias*, Séptima edición, Cengage Learning Editores, página 298

For each clinical trial the main variable of postural balance is the area of the center of pressure COP demonstrated in previous studies. (Gatica et al., 2010[f] y Gatica et al., 2016[c,d]).

Clinical Trial 1:

Neurology area group: patients with cerebral palsy type hemiplegia.

Assuming an alpha risk of 0.05 and a beta risk of 0.2 in a two-sided contrast, 20 subjects in the first group/arm and 20 in the second are required to detect a maximum difference equal to or greater than 4.6 units.

A common standard deviation of 4.974 is assumed. A rate of loss to follow-up of 5% has been estimated.

The n calculated to carry out this stage is 20 patients with CP for each version/branch of therapy (face-to-face and remote). For the remote version they will be distributed randomly and balanced.

Clinical trial 2:
Older Adults

Assuming an alpha risk of 0.05 and a beta risk of 0.2 in a two-sided contrast, 8 subjects in the first group/arm and 8 in the second are required to detect a difference equal to or greater than 1.5 units.

A common standard deviation of 1 is assumed. A rate of loss to follow-up of 5% has been estimated.
The n calculated to carry out this stage is 8 OA for each version/branch of therapy (face-to-face and remote).

**Randomization and recruitment:**
Simple and age-paired randomization will be performed in this study, for each trial (stage 1 and 2). Randomization is the process by which subjects are randomly assigned to different study groups at the start of the trial (face-to-face and remote). There are many randomization methods of different complexity, but they all follow a fundamental and simple principle: where each patient in the study population has a fixed and known probability of being selected. The fundamental reason is the need to avoid selection bias, whereby an asymmetry in the forecast of the branches in comparison can occur.
Randomization generates groups comparable to each other, both in known and unknown factors, in such a way that the only difference between the groups under study will be the intervention, guaranteeing the validity of the statistical significance tests. For this, each study population (stage 1 and 2) will be incorporated into the SPSS software, through an identification ID and without a pre-established order. In this way, two random samples will be drawn (face-to-face and distance version).
Recruitment will be made by telephone call from the records and contacts in

the respective centers. This will be done by an independent professional (MEA).

-Study variables for both clinical trials:

Variable Result, is the balance/postural balance evaluated in a standing position. This is a construct which will be measured with the force platform and the balance board, generating the center of pressure or COP. The following continuous variables are obtained from the COP:

Primary variable: COP area or also called COPSway.

Secondary variables: speed and sway or also known by the name of standard deviation (in its abbreviation in English SD-COP), both in the M-L (X axis) and A-P (Y axis) directions.

Exposure Variable, is the "version of the exercise program with NWBB", face to face or remotely.

**-Demographic and clinical characteristics of the sample:** sex, age, weight, height, history of falls, drug use and history of metabolic diseases (diabetes and hypertension).

**-Procedures:** Balance/postural balance assessments will be performed at the start and end of the exercise program workouts with NWBB for the distance version and with a force platform for the face-to-face version, at the LCMH. Standing on the platform, the balance will be evaluated both in eyes open and closed conditions (30 s/each condition). Each version of the exercise program with NWBB will include a total of 18 sessions, 3 times a week, for 6 weeks (Gatica et al., 2010, Gatica-Rojas et al., 2016[c,d]).

**-Data collection techniques:** measurements will be recorded in a file for each participant. The data will be recorded in a database in Excel format by an external trained professional (MEA), avoiding measurement bias.

It is a procedure of this research team that the data collected from the participants during the research will be available to the neurology centers to be incorporated into the respective clinical records and club for the elderly. In addition to delivering a written report and a personalized or individual (not group) verbal explanation to each participant in this investigation.

- **Statistical methods:** Shapiro Wilks test will be used to measure the normality of the data for each measurement variable and each group/branch of study. The demographic and clinical characteristics of the participants in each group will be compared using Fisher's exact test or Chi square or t test for independent samples. To compare the effect of therapist-guided therapy in rehabilitation centers versus families at home, the t test for independent samples or its non-parametric counterpart will be performed. To compare the effects of VR exercise programs between the face-to-face and telerehabilitation versions, a t test for independent samples and a linear

regression analysis will be performed. The statistical significance level will be $p<0.05$.
Analysis by intention to treat will be carried out for the missing data that the evaluations may present (point where the data is obtained). According to the percentages of data loss <3%, it will be carried out with the average of the data corresponding to the variable that corresponds to be imputed, in the event that the losses are >3%, statistical imputations will be made according to the behavior of data loss.

**-Clinical trial in international registries:** When advancing and guaranteeing the execution of phase 3 of this project (clinical trials), the respective clinical trials will be registered within the same registry in the Australian New Zeeland Clinical Trials Registry: http://www.anzctr.org. au/

**Ethical discussion:**
The procedure described in this project, as well as the informed consent documents, have been prepared and respecting what was declared in Helsinki. Therefore, this research establishes clear protocols where there are no risks to the health of the participants, well-being is above science, and voluntariness will be respected at all times. In line with this, the participants can withdraw at any time from this project, and their withdrawal will not constitute a negative effect on it or on the public attention services or Clubs, respectively that they belong to.
Informed consent is a process of verbal and written (non-technical) explanation. The verbal explanation refers to a conversation between the responsible researcher (General Director) and the participant (in the case of children-adolescents it will be given in the company of their parents/legal guardians), in which the procedures in which which ones will participate The written part will be through a document called informed consent, which must be signed by the researcher and the participant. In the case of children-adolescents, the written section will be a simple document (1 page) that will describe the general procedures complemented by the verbal explanation (with diagrams), added to them, the parents/legal guardians will receive a written informed consent.

The direct benefits are those concerning the research proposal itself and aimed at the health of the participants (improving balance and quality of life), in addition to providing free of charge all the necessary technology to allow telerehabilitation (software, consoles, computers , data and 6 months of internet connection if necessary; depending on what each user requires). No other benefit is identified, that is, you will not receive financial payment for participating in this project. The confidentiality of all the data obtained is guaranteed, as well as anonymity in the event of its disclosure in scientific

media.

The information obtained during the execution of this project will be stored in a personal computer of the Director General, assigned by the University of Talca, with a secret access code. A backup copy of the information (data) will be kept on an external hard drive belonging to the Director General which is kept under lock and key in his office. This information will be stored for 2 years after the end of the project, after which it will be destroyed. The informed consents that will be applied in paper format will be stored under lock and key in a wooden cabinet (sheet metal). The keys (2), as well as access to this piece of furniture, are managed only by the General Director. The furniture will be located in the premises of the Human Motor Control Lab with access restricted to authorized personnel.

**References:**

[a]**Gatica** VR, Rebolledo GM, Muñoz EG, Cortés NI, Gaete CB, Delgado CM. Differences in standing balance between patients with diplegic and hemiplegic cerebral palsy. Neural Regen Res. 2013 Sep 15;8(26):2478-83. doi: 10.3969/j.issn.1673-5374.2013.26.009.

[b]**Gatica** VF, Irene Velásquez S, Méndez GA, Guzmán EE, Manterola CG. Differences in standing balance in patients with cerebral palsy and typically developing children. Biomedica. 2014 Jan-Mar;34(1):102-9.

[c]**Gatica-Rojas**, V et al. Change in functional balance after an exercise programme with Nintendo Wii in Latino patients with cerebral palsy: a case series. J. Phys. Ther. Sci. 28: 2414–2417, 2016

[d]**Gatica-Rojas**, V et al. Does Nintendo Wii Balance Board improve standing balance? A randomised controlled trial in children with cerebral palsy. Eur J Phys Rehabil Med 2016 (aceptado)

[e]**Gatica-Rojas** V, Méndez-Rebolledo G. Virtual reality interface devices in the reorganization of neural networks in the brain of patients with neurological diseases. Neural Regen Res. 2014 Apr 15;9(8):888-96.

[f]**Gatica** RV, Elgueta CE, Vidal SC, Cantin ML, Fuentealba JA. Impacto del entrenamiento del balance a través de realidad virtual en una población de

adultos mayores. Int J Morphol 2010;28:303-308.

[g]**Gatica-Rojas**, V et al. Correlation between centre of pressure and functional balance tests in nonfallers elderly practitioners of Tai Chi Chuan. J. Phys. Ther. Sci. 28: 2350–2352, 2016

Truter P, Russell T, Fary R. The validity of physical therapy assessment of low back pain via telerehabilitation in a clinical setting. Telemed J E Health. 2014;20(2):161-7. doi: 10.1089/tmj.2013.0088.

Mani S, Sharma S, Omar B, Paungmali A, Joseph L. Validity and reliability of Internet-based physiotherapy assessment for musculoskeletal disorders: A systematic review. J Telemed Telecare. 2016 Mar 31. pii: 1357633X16642369. [Epub ahead of print]

Richardson BR, Truter P, Blumke R, Russell TG. Physiotherapy assessment and diagnosis of musculoskeletal disorders of the knee via telerehabilitation. J Telemed Telecare. 2016 Mar 15. pii: 1357633X15627237. [Epub ahead of print]

Veras M, Kairy D, Rogante M, Giacomozzi C, Saraiva S. Scoping review of outcome measures used in telerehabilitation and virtual reality for post-stroke rehabilitation. J Telemed Telecare. 2016 Jun 24. pii: 1357633X16656235. [Epub ahead of print]

Cottrell MA, Galea OA, O'Leary SP, Hill AJ, Russell TG. Real-time telerehabilitation for the treatment of musculoskeletal conditions is effective and comparable to standard practice: A systematic review and meta-analysis. Clin Rehabil. 2016 May 2. pii: 0269215516645148. [Epub ahead of print]

Boyd RN, Baque E, Piovesana A, Ross S, Ziviani J, Sakzewski L, et al. Mitii™ ABI: study protocol of a randomised controlled trial of a web-based multi-modal training program for children and adolescents with an Acquired Brain Injury (ABI). BMC Neurol. 2015;15:140. doi: 10.1186/s12883-015-0381-6.

Cans C. Surveillance of cerebral palsy in Europe: A collaboration of cerebral palsy surveys and registers. Dev Med Child Neurol. 2000; 42:816-24.

Johnson A. Prevalence and characteristics of children with cerebral palsy in Europe. Dev Med Child Neurol. 2002 Sep;44(9):633-40.

Tousignant M, Moffet H, Boissy P, Corriveau H, Cabana F, Marquis F. A

randomized controlled trial of home telerehabilitation for post-knee arthroplasty. J Telemed Telecare. 2011;17(4):195-8. doi: 10.1258/jtt.2010.100602.

Tousignant M, Boissy P, Corriveau H, Moffet H. In home telerehabilitation for older adults after discharge from an acute hospital or rehabilitation unit: A proof-of-concept study and costs estimation. Disabil Rehabil Assist Technol. 2006 Sep;1(4):209-16.

Shkuratova N, Taylor N. The influence of age on gait parameters during the transition from a wide to a narrow pathway. Physiother Res Int. 2008 Jun;13(2):75-83.

Steindl R, Kunz K, Schrott-Fischer A, Scholtz A. Effect of age and sex on maturation of sensory systems and balance control. Dev Med Child Neurol. 2006;48:477-82.

Clark RC, Saxion CE, Cameron KL, Gerber JP. Associations between three clinical assessment tools for postural stability. N Am J Sports Phys Ther. 2010 Sep;5(3):122-30.

Fondo Nacional de la Discapacidad, FONADIS – Instituto Nacional de Estadística, INE. (2004). Informe Ejecutivo VII Región del Maule. 1º Estudio Nacional de la Discapacidad en Chile.

Casen 2013. http://www.senama.cl/n5729_15-03-2015.html

## III. Gantt Chart[3]

| Ítem | Semestre 1 | | | | | | Semestre 2 | | | | | | Semestre 3 | | | | | | Semestre 4 | | | | | |
|---|---|---|---|---|---|---|---|---|---|---|---|---|---|---|---|---|---|---|---|---|---|---|---|---|
| Desarrollo de software etapa Alfa | | | | | | X | X | X | X | | | | | | | | | | | | | | | |
| Desarrollo de software etapa Beta | | | | | | | | | X | X | X | X | | | | | | | | | | | | |
| Difusión en prensa y web | | | | | | | | | | | X | X | | | | | | | | | | | | |
| Reclutamiento de pacientes/participantes | | | | | | | | | | | | | | | | X | X | X | X | X | | | | |
| Capacitación y calibración de monitores | | | | | | | | | | | X | X | X | X | X | | | | | | | | | |
| Prueba de plataforma en centros | | | | | | | | | | | | | X | X | X | | | | | | | | | |
| Prueba de plataforma en hogares | | | | | | | | | | | | | | X | X | X | X | | | | | | | |
| Desarrollo de la intervención | | | | | | | | | | | | | | | | | X | X | X | X | X | | | |
| Gira tecnológica | | | | | | | | | | | | | | | | | | | | | | X | X | |
| Análisis de datos | | | | | | | | | | | | | | | | | | | | | | X | X | |
| Preparación de informes | X | X | X | X | X | X | X | X | X | X | X | X | X | X | X | X | X | X | X | X | X | X | X | X |
| Difusión de resultados a comunidad | | | | | | | | | | | | | | | | | | | | | | | X | X |
| Difusión científico tecnológica | | | | | | | | | | | | | | | | | | | | | X | X | X | X |
| Cierre del proyecto. | | | | | | | | | | | | | | | | | | | | | | | | X |

[3] Elabore una carta Gantt del proyecto de acuerdo a su configuración proyectada.
